# Adipose Mesenchymal Stromal Cell-Derived Exosomes Prevent Testicular Torsion Injury *via* Activating PI3K/AKT and MAPK/ERK1/2 Pathways

**DOI:** 10.1155/2022/8065771

**Published:** 2022-06-16

**Authors:** Hengchen Liu, Manyu Shi, Xiangqi Li, Wenjun Lu, Mingzhao Zhang, Tingting Zhang, Yang Wu, Zenan Zhang, Qingbo Cui, Shulong Yang, Zhaozhu Li

**Affiliations:** Department of Pediatric Surgery, The Second Affiliated Hospital of Harbin Medical University, No. 246, Xuefu Road, Nangang District, Harbin 150001, China

## Abstract

Adipose mesenchymal stromal cell-derived exosomes (ADSC-Exos) have shown great potential in the treatment of oxidative stress induced by ischemia-reperfusion injury. However, alleviation of testicular torsion injury by ADSC-Exos has not been reported. Therefore, we investigated the protective effect of ADSC-Exos against testicular torsion-detorsion injury. ADSC-Exos were isolated by ultracentrifugation and injected into torsion-detorsion-affected testes of rats. H&E staining and sperm quality were used to evaluate the therapeutic effects of ADSC-Exos, and tissue oxidative stress was measured by determining MDA and SOD levels. In addition, TUNEL staining and immunohistological analysis (Ki67, Cleaved Caspase-3, IL-6, IL-10, CCR7, and CD163) were used to clarify the effects of ADSC-Exos on spermatogenic cell proliferation, apoptosis, and the inflammatory microenvironment *in vivo*. Possible signaling pathways were predicted using sequencing technology and bioinformatics analysis. The predicted signaling pathways were validated *in vitro* by assessing the proliferation (EdU assay), migration (transwell assay and scratch test), and apoptosis (flow cytometry, TUNEL staining, and western blotting) of spermatogenic cells. The results showed that ADSC-Exos alleviated testicular torsion-detorsion injury by attenuating oxidative stress and the inflammatory response. In addition, ADSC-Exos promoted the proliferation and migration of spermatogenic cells and inhibited their apoptosis by activating the PI3K/AKT and MAPK/ERK1/2 signaling pathways.

## 1. Introduction

Testicular torsion is a urological emergency characterized by acute scrotal pain with nausea and vomiting, which often occurs in male children [1, 2]. The incidence of testicular torsion in adolescents is 1/4000, accounting for 26% of scrotal pain cases [3]. Early detection, diagnosis, and treatment are key to avoiding testicular necrosis [4, 5]. However, 10% of testicles cannot be saved even in the first 6 h after torsion occurs [6]. In addition, the rate of testicular atrophy and infertility is reportedly between 40% and 60% despite successful surgical intervention [7].

The primary pathological mechanism of testicular torsion is ischemia-reperfusion (I/R) injury. Changes in microvascular blood flow cause the release of proinflammatory cytokines and production of large amounts of reactive oxygen species (ROS), thus causing membrane lipid peroxidation [7]. Polyunsaturated fatty acids, which are major components of the sperm cell membrane, are highly susceptible to ROS-induced damage [8]. In addition, ROS production affects spermatogenic cell structure and function or even causes apoptosis, leading to spermatogenic impairment [9, 10]. Recently, several antioxidants, such as *Fumaria parviflora* extract, vitamin C, and coenzyme Q10, have been shown to help to reduce ROS levels and improve sperm parameters [8, 11]. Thus, early surgery combined with antioxidants, anti-inflammatory cytokines, or other drugs is important means of improving the prognosis of testicular torsion.

Adipose-derived mesenchymal stromal cells (ADSCs) play profound roles in various preclinical studies [12, 13]. For example, abundant data have demonstrated that the anti-inflammatory, antioxidant, and antiapoptotic effects of ADSCs are helpful in the treatment of organ I/R injury [14–17]. Although local injection of ADSCs has been shown to rescue testicular torsion-induced infertility [18, 19], studies have found that transplanted mesenchymal stromal cells (MSCs) do not survive effectively in the ischemic microenvironment post infarction [20]. Therefore, the functional benefits of transplanted MSCs are likely due to the release of paracrine mechanisms, such as exosomes, which can regulate cell growth [21]. Exosomes derived from ADSCs (ADSC-Exos) have been shown to effectively reduce I/R injury in the brain [22], heart [23], and kidney [16]. In addition, ADSC-Exos have shown promising efficacy in the treatment of erectile dysfunction caused by conditions such as diabetes mellitus and postradical prostatectomy [24, 25]. In a recent study, Bader et al. demonstrated that cell culture medium containing ADSC-Exos could improve sperm parameters in a concentration- and time-dependent manner [26]. However, alleviation of testicular I/R injury by ADSC-Exos has not been reported. Therefore, the aim of this study was to evaluate the effects of ADSC-Exos on spermatogenic cell viability, sperm quality, and inflammation *in vivo* and the proliferation, migration, and apoptosis of spermatogenic cells *in vitro*. In addition, we aimed to identify the major pathways through which ADSC-Exos exert their effects by microRNA (miRNA) sequencing and bioinformatics analysis.

## 2. Material and Methods

### 2.1. Animals

Male Sprague-Dawley rats (200–250 g, 8–10 weeks of age) were purchased from the Animal Experiment Center of Harbin Medical University. All experiments involving animals were approved by the Ethics Committee of Harbin Medical University (approval no. Ky2018-135).

### 2.2. Isolation and Identification of ADSCs and ADSC-Exos

ADSCs were isolated from the subcutaneous fat of rats, which was sliced into 1 mm^3^ sections and digested in 0.2% collagen I for 1 h at 37°C. Next, samples were centrifuged at 1000 × *g* for 10 min, and the fatty layer and supernatant were removed. The cells obtained in the pellet were cultured in DMEM/F12 (Sigma-Aldrich, St. Louis, MO, USA) supplemented with 10% FBS (Sigma-Aldrich), 1% penicillin, and streptomycin (Beyotime Biotech, Haimen, China) at 37°C under 5% CO_2_. At passage three, the culture medium was replaced with DMEM/F12 supplemented with 10% exosome-depleted FBS (Sigma-Aldrich), and cells were incubated for 24 h. Finally, the ADSCs were collected, and ADSC-Exos were isolated by ultracentrifugation, as previously reported [27]. The surface markers (CD90, CD105, CD34, CD45, and CD11b) and multilineage differentiation capacity (adipogenic, osteogenic, and chondrogenic) of ADSCs were previously reported [27]. Nanoparticle tracking analysis, transmission electron microscopy (TEM), and western blotting were used to identify the collected exosomes.

### 2.3. Experimental Protocols and Surgical Procedures

Sixty rats were randomly divided into five groups (*n* = 12): Group 1 animals underwent surgery for scrotal incision and suturing (Control); Group 2 animals underwent surgery for testicular torsion, received a local injection of 100 *μ*L PBS before detorsion, and were sacrificed 3 days later (I/R-3D); Group 3 animals underwent surgery for testicular torsion, received a local injection of 100 *μ*L PBS containing 400 *μ*g ADSC-Exos before detorsion, and were sacrificed 3 days later (ADSC-Exos-3D); Group 4 animals underwent surgery for testicular torsion, received a local injection of 100 *μ*L PBS before detorsion, and were sacrificed 7 days later (I/R-7D); and Group 5 animals underwent surgery for testicular torsion, received a local injection of 100 *μ*L PBS containing 400 *μ*g ADSC-Exos before detorsion, and were sacrificed 7 days later (ADSC-Exos-7D).

All surgical procedures were performed under aseptic conditions with ketamine-based anesthesia (50 mg/kg). To achieve unilateral testicular torsion, the testis and spermatic cord were exposed through a left inguinal incision, and the left testis was rotated counterclockwise 720° for 3 h [18, 28]. After the testis was fixed to the tunica albuginea with 6/0 silk sutures, the incision was closed with 4/0 silk sutures, and the operated testis was protected with wet gauze and warm light for 3 h. Half an hour before the detorsion procedure, 100 *μ*L ADSC-Exos or PBS was injected into the testis. Given that Cui et al. confirmed that 400 *μ*g ADSC-Exos can protect the myocardium from I/R injury in Sprague-Dawley rats [13], the same dose was selected for the rat testicular torsion-detorsion injury model in the current study. Subsequently, the testis underwent detorsion using the same surgical approach. The testis was fixed in the normal anatomical position with 6-0 silk sutures, and the incision was closed with 4-0 silk sutures. All experimental animals were treated with 0.02 mg/kg buprenorphine for pain relief. Animals were sacrificed on the third and seventh postoperative days, and the testes and epididymides were collected for further study.

### 2.4. Determination of Spermatozoal Parameters

Rat epididymal tissues were cut into 1 mm^3^ cubes and immersed in 0.9% NaCl at 37°C for 20 min to extract the spermatozoa. Sperm quality (quantity, morphology, and motility) was assessed using the WHO sperm analysis method [29]. The morphology and motility of 200 sperm in each group were evaluated.

### 2.5. Histopathological and Immunohistological Analyses

The testicular tissues were fixed in Davidson's fixative (Beyotime Biotech), and tissue sections were stained with hematoxylin and eosin (H&E). Johnsen's score (Table 1) was used to evaluate spermatogenic function [30]. Fifty seminiferous tubules were examined in each testis.

The tissue sections were immunohistochemically stained using the primary antibody anti-Ki-67 (AF0198; Affinity Biologicals, Ancaster, ON, Canada), followed by incubation with goat anti-rabbit IgG secondary antibody (ab6721; Abcam, Cambridge, UK). For immunofluorescence analysis, the tissue sections were incubated with primary antibodies anti-Cleaved Caspase-3 (AF7022; Affinity Biologicals), anti-CCR7 (ab32527; Abcam), anti-CD163 (ab182422; Abcam), anti-IL-6 (TA500067S; Origene), and anti-IL-10 (ab33471; Abcam), followed by incubation with secondary antibodies (SA00013; ProteinTech, Chicago, IL, USA). Nuclei were stained with 4′,6-diamidino-2-phenylindole (DAPI). Positive signals were quantified using the ImageJ software.

### 2.6. Biochemical Analysis

The malondialdehyde (MDA) content in testicular tissue was determined by colorimetry using the Lipid Peroxidation MDA Assay Kit (Beyotime Biotech). Superoxide dismutase (SOD) activity in testicular tissue was detected using the CuZn/Mn-SOD Assay Kit with WST (Beyotime Biotech).

### 2.7. Cell Culture and Treatment

The GC-1 spg cell line was purchased from the American Type Culture Collection (ATCC, Manassas, VA, USA). The cells were cultured in DMEM with high glucose (Sigma-Aldrich) supplemented with 10% FBS and 1% penicillin and streptomycin at 37°C with 5% CO_2_. To establish the I/R model *in vitro*, 1 × 10^6^ cells were cultured in glucose-free DMEM (Sigma-Aldrich) in a 1% O_2_ environment for 18 h, followed by reoxygenation with normal O_2_ in complete medium with or without exosomes.

*In vitro*, GC-1 spg cells were randomly sorted into six groups as follows: Group 1 cells were cultured under normal conditions (Control); Group 2 cells underwent cellular I/R injury only (I/R); Group 3 cells underwent cellular I/R injury, followed by reoxygenation with 100 *μ*g/mL ADSC-Exos (ADSC-Exos (100)); Group 4 cells underwent cellular I/R injury, followed by reoxygenation with 200 *μ*g/mL ADSC-Exos (ADSC-Exos (200)); Group 5 cells were pretreated with 50 *μ*M LY294002 (PI3K/AKT inhibitor; MedChemExpress, Monmouth Junction, NJ, USA) for 30 min before reoxygenation with 200 *μ*g/mL ADSC-Exos (ADSC-Exos+LY); and Group 6 cells were pretreated with 50 *μ*M PD98059 (MAPK/ERK1/2 inhibitor; MedChemExpress) for 30 min before reoxygenation with 200 *μ*g/mL ADSC-Exos (ADSC-Exos+PD). The exosome dose was selected based on a previous study [31]. Cells from each group were collected after 30 min or 24 h for western blotting, after 3 h for flow cytometric analysis or TUNEL assay, and after 24 h for EdU, transwell, and scratch assays.

### 2.8. ADSC-Exos Internalization Analysis

ADSC-Exos were labeled using 1 *μ*M PKH26 dye (Sigma-Aldrich) in Diluent C for 5 min. After ultracentrifugation, PKH-26-labeled ADSC-Exos were added to GC-1 spg cells cultured in exosome-depleted medium. Nuclei were counterstained with DAPI after 24 h, and ADSC-Exos internalization was observed under a fluorescence microscope.

### 2.9. Proliferation of GC-1 spg Cells

For cell proliferation analysis, 10 *μ*M 5-ethynyl-2-deoxyuridine (EdU) was added to GC-1 spg cells for 30 min. Subsequently, the cells were fixed and stained using an EdU assay kit (UE, China). Cell proliferation was observed under a fluorescence microscope after nuclei were counterstained with DAPI.

### 2.10. Migration of GC-1 spg Cells

For cell migration analysis, scratch tests and transwell assays were performed. For the scratch test, a scratch was made through the cultured cells after hypoxic injury. The extent of cell migration was measured after 0 and 24 h.

The transwell assay was conducted after hypoxic injury. A total of 1 × 10^5^ GC-1 spg cells were cultured in the upper chamber. Reoxygenation medium was added to the lower chamber. After 24 h, cells in the upper chamber were fixed with paraformaldehyde and stained with crystal violet. The degree of cell migration was determined by counting the number of cells in the upper chamber under a light microscope.

### 2.11. Western Blot Analysis

Cells were lysed in RIPA buffer (Beyotime Biotech) to extract the proteins. Immunoblotting was performed with primary antibodies anti-Hsp70 (ab2787; Abcam), anti-TSG101 (ab125011; Abcam), anti-CD9 (ab92726; Abcam), anti-AKT (4685S; Cell Signaling Technology, Danvers, MA, USA), anti-phospho-AKT (4060S; Cell Signaling Technology), anti-ERK1/2 (4695S; Cell Signaling Technology), anti-phospho-ERK1/2 (4370S; Cell Signaling Technology), anti-Bcl-2 (ab196495; Abcam), anti-Bax (ab32503; Abcam), and anti-*β*-actin (ab8226; Abcam), according to the manufacturer's instructions. Goat anti-rabbit IgG (ab205718; Abcam) was used as the secondary antibody. The quantification of protein bands was performed using the ImageJ software.

### 2.12. Flow Cytometry Analysis

To analyze apoptosis, cells were collected and stained using the FITC Annexin V Apoptosis Detection Kit (Becton-Dickinson, Franklin Lakes, NJ, USA) and then analyzed by flow cytometry. The data were analyzed using the FACSDiva software (Becton-Dickinson).

### 2.13. Terminal Deoxynucleotidyl Transferase dUTP Nick End Labeling (TUNEL) Assay

The apoptosis of spermatogenic cells *in vivo* was detected using a TUNEL apoptosis assay kit (Wanleibio, Shenyang, China). Briefly, paraffin sections of testicular tissues were incubated with 50 *μ*L TUNEL reaction mixture. The sections were dehydrated and fixed after nuclei were counterstained with hematoxylin. Apoptosis of spermatogenic cells was evaluated under a light microscope.

In addition, the TUNEL assay was performed *in vitro*. Briefly, GC-1 spg cells from each group were fixed and stained using the TUNEL Assay Apoptosis Detection Kit (UE). Apoptosis was observed under a fluorescence microscope after nuclei were counterstained with DAPI.

### 2.14. miRNA Sequencing and Data Analysis

miRNA sequencing of ADSC-Exos was performed by the OE Biotech Company (Shanghai, China). Briefly, 20 ng of exosomal RNA was extracted and sequenced using the HiSeq 2500 system (Illumina, San Diego, CA, USA) (*n* = 3). Target genes of the top 50 highly expressed miRNAs in ADSC-Exos were predicted using the miRanda software. Gene Ontology (GO) and Kyoto Encyclopedia of Genes and Genomes (KEGG) pathway enrichment analyses of the target genes were performed using DAVID (https://david.ncifcrf.gov/) and KOBAS 3.0 (http://kobas.cbi.pku.edu.cn/kobas3/), respectively. The results were visualized using the R software.

### 2.15. Statistical Analysis

Data are expressed as the mean ± standard deviation (SD). Statistical analysis for multiple groups was conducted using the Tukey-Kramer *t*-test. *P* < 0.05 was considered statistically significant.

## 3. Results

### 3.1. Characterization of ADSC-Exos

**Figure 1:**
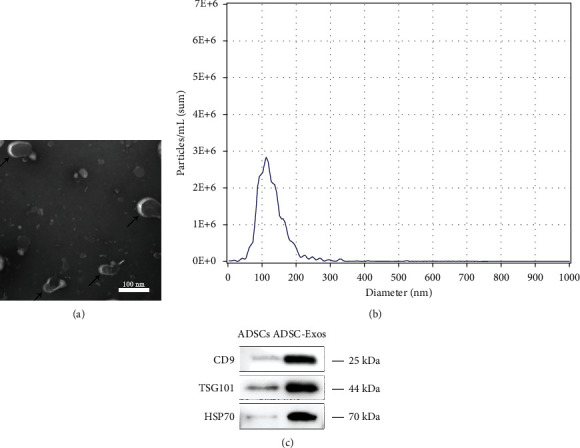
Characterization of ADSC-Exos. (a) Morphology of ADSC-Exos under a transmission electron microscope. (b) Particle size distribution. (c) Western blot was used to detect exosome surface markers. Bars, 100 nm.

ADSC-Exos exhibited circular vesicular structures under TEM (Figure 1(a)). The average size of ADSC-Exos was 111.9 nm (Figure 1(b)). Western blot analysis demonstrated high expression of ADSC-Exos surface markers, including CD9, TSG101, and HSP70 (Figure 1(c)).

### 3.2. Alleviation of Testicular Torsion-Detorsion Injury by ADSC-Exos

**Figure 2:**
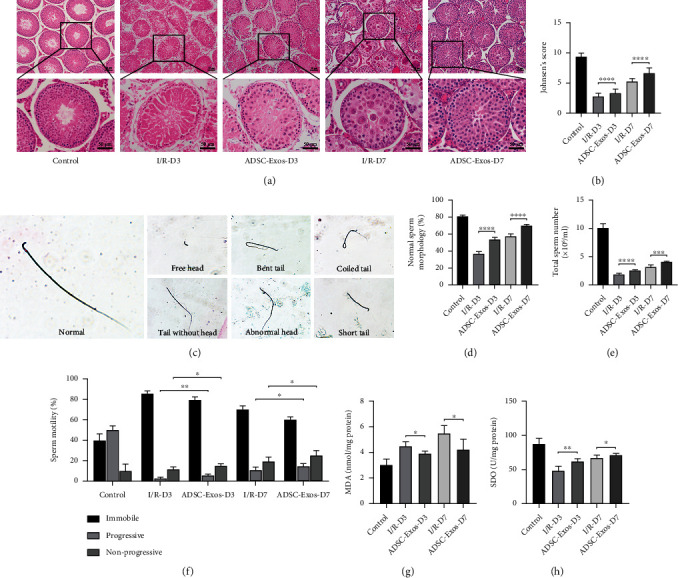
ADSC-Exos alleviate testicular torsion-detorsion injury. (a, b) H&E staining after torsion-detorsion injury at days 3 (*n* = 6) and 7 (*n* = 6). (c) Sperm with normal and abnormal morphology. (d–f) Results of sperm parameters (quantity, morphology, and motility) at days 3 (*n* = 6) and 7 (*n* = 6). (g, h) Results of biochemical analysis (MDA and SOD) at days 3 (*n* = 6) and 7 (*n* = 6). Bars, 50 *μ*m. Data are represented as mean ± SD. ^∗^*P* < 0.05, ^∗∗^*P* < 0.01, ^∗∗∗^*P* < 0.001, ^∗∗∗∗^*P* < 0.001.

H&E staining showed that testes in the Control group exhibited normal testicular structure and seminiferous tubule morphology, as well as many mature sperm. However, severe damage was observed in the testes three days after torsion-detorsion injury, which manifested as seminiferous tubule disorder, unclear boundaries, interstitial edema, and few sperm. In contrast, the histological appearance of the testes was significantly improved after treatment with ADSC-Exos. Seven days after torsion-detorsion injury, the ADSC-Exos group had significantly more spermatogenic cells than the I/R group, and the cells exhibited a more orderly arrangement (Figure 2(a)). Spermatogenic function was substantially improved in the ADSC-Exos group on days 3 and 7 (Figure 2(b)).

To further determine whether ADSC-Exos could improve spermatogenesis after torsion-detorsion injury, sperm was extracted from epididymides in each group. Analysis of sperm parameters indicated that testicular torsion-detorsion injury led to poor sperm quality. Sperm quantity, mobility, and morphology were significantly decreased in the I/R groups compared to those in the Control group. However, treatment with ADSC-Exos significantly improved sperm quality (Figures 2(c)–2(f)). Furthermore, MDA levels in the I/R groups were significantly increased compared to those in the Control group, while SOD levels were decreased. ADSC-Exos treatment reduced MDA levels and increased SOD levels compared to those in the I/R groups (Figures 2(g) and 2(h)). Spermatogenic function and sperm quality did not differ in the contralateral testis among the three treatment groups on day 7 (Figure S1).

### 3.3. Protection of Spermatogenic Cell Activity by ADSC-Exos

**Figure 3:**
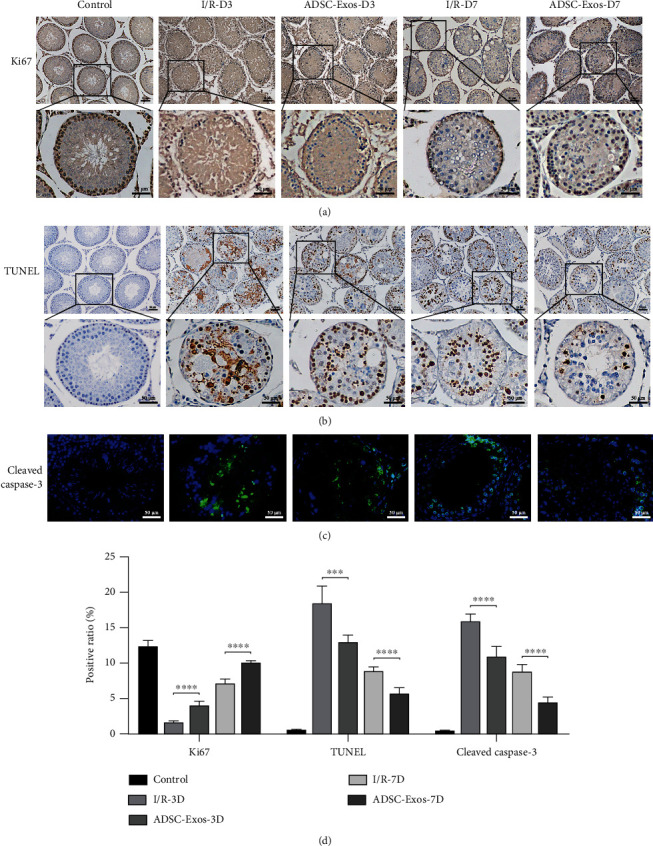
ADSC-Exos promote spermatogenic cell proliferation and inhibit apoptosis in vivo. (a) The expression of Ki67 was detected by immunohistochemistry at days 3 (*n* = 6) and 7 (*n* = 6). (b) TUNEL assay of testicular tissues after torsion-detorsion injury at days 3 (*n* = 6) and 7 (*n* = 6). (c) The expression of Cleaved Caspase-3 was detected by immunofluorescence at days 3 (*n* = 6) and 7 (*n* = 6). (d) Quantitative analysis of Ki67, TUNEL, and Cleaved Caspase-3 expression. Bars, 50 *μ*m. Data are represented as mean ± SD. ^∗∗∗^*P* < 0.001, ^∗∗∗∗^*P* < 0.001.

Immunohistochemical analysis revealed that the expression of Ki67 in spermatogenic cells was decreased after testicular torsion-detorsion injury. However, treatment with ADSC-Exos significantly increased the number of Ki67^+^ spermatogenic cells (Figure 3(a)). TUNEL staining demonstrated massive spermatogenic cell apoptosis after testicular torsion-detorsion injury. However, the numbers of apoptotic spermatogenic cells in the ADSC-Exos groups on days 3 and 7 were lower than those in the I/R groups (Figure 3(b)). As expected, the immunofluorescence staining results for Cleaved Caspase-3 (apoptosis marker) concurred with the TUNEL staining results (Figure 3(c)). These results indicated that ADSC-Exos promoted spermatogenic cell proliferation and reduced apoptosis after testicular torsion-detorsion injury (Figure 3(d)).

### 3.4. miRNA Sequencing and Bioinformatics Analysis of ADSC-Exos

**Figure 4:**
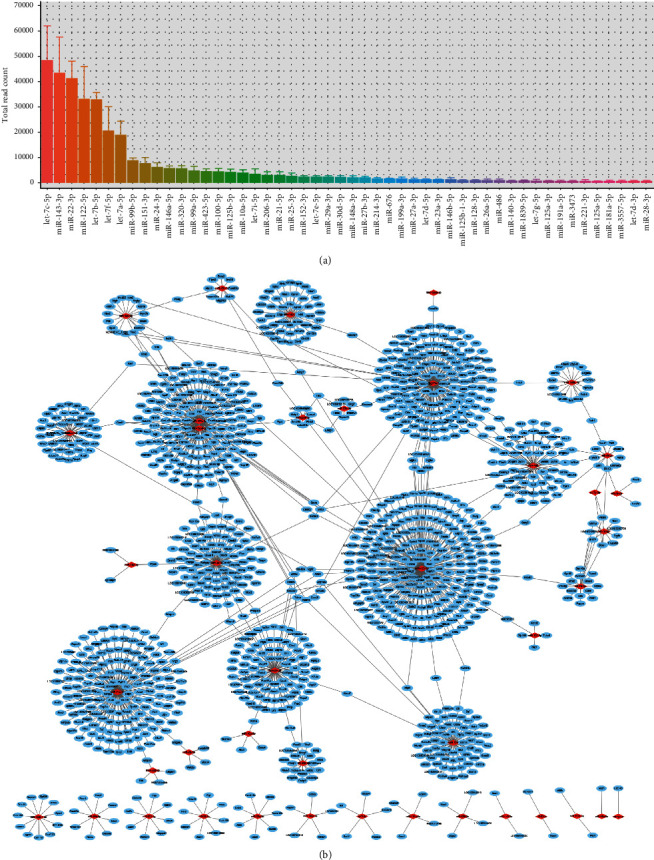
miRNA sequencing and target gene analysis of ADSC-Exos. (a) The top 50 miRNAs detected in ADSC-Exos (*n* = 3). (b) Prediction of miRNA target genes.

**Figure 5:**
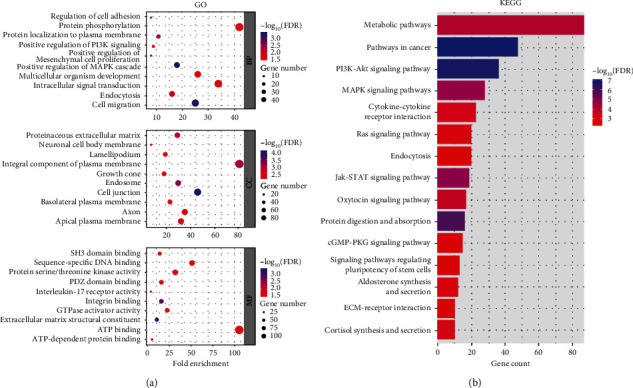
Bioinformatics analysis of ADSC-Exos target genes. (a) GO and (b) KEGG pathway enrichment analyses of the possible target genes.

The top 50 miRNAs detected in ADSC-Exos are shown in Figure 4(a). To ascertain their possible target genes, GO and KEGG pathway enrichment analyses were performed (Figure 4(b)). The biological process (BP) was mainly enriched in “regulation of cell adhesion,” the cellular component (CC) was mainly enriched in “proteinaceous extracellular matrix,” and the molecular function (MF) was mainly enriched in “SH3 domain binding” (Figure 5(a)). KEGG pathway enrichment analyses indicated that the PI3K/AKT and MAPK signaling pathways were the main signaling pathways through which miRNAs in ADSC-Exos function (Figure 5(b)). Therefore, the hypothesis that ADSC-Exos alleviate testicular torsion-detorsion injury *via* the PI3K/AKT and MAPK/ERK1/2 signaling pathways was further investigated.

### 3.5. ADSC-Exos Activate the PI3K/AKT and MAPK/ERK1/2 Signaling Pathways

**Figure 6:**
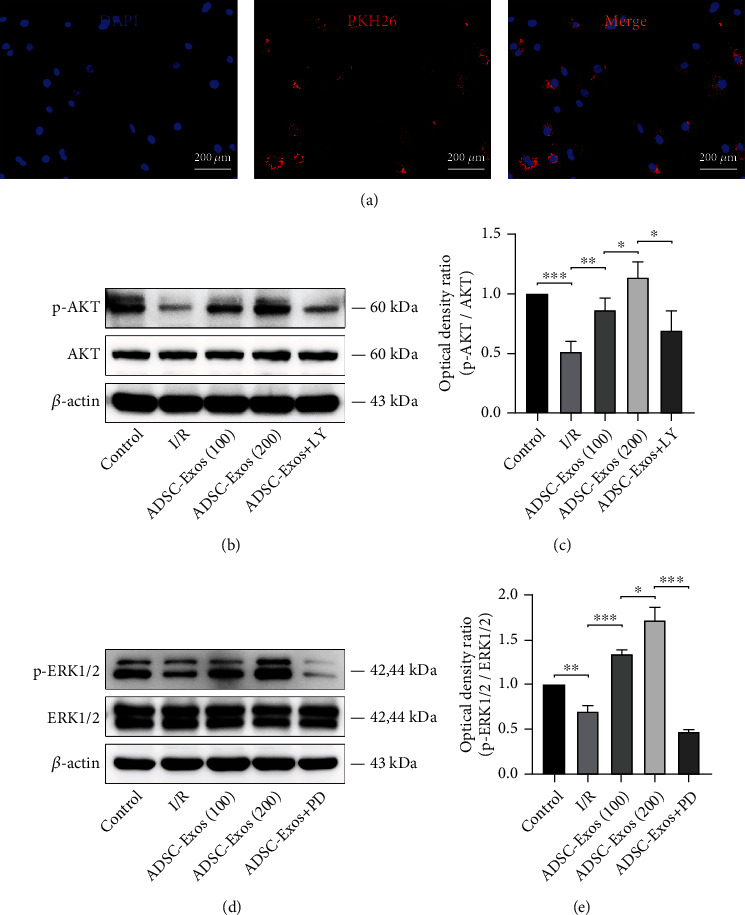
ADSC-Exos activate PI3K/AKT and MAPK/ERK1/2 signaling pathways in GC-1 spg cells. (a) PKH26-labeled ADSC-Exos internalization by GC-1 spg cells. (b–e) Western blot analysis of protein levels of p-AKT and p-ERK induced by different concentrations of ADSC-Exos or pathway inhibitors. Bars, 200 *μ*m. Data are represented as mean ± SD. ^∗^*P* < 0.05, ^∗∗^*P* < 0.01, ^∗∗∗^*P* < 0.001.

PKH-26-labeled ADSC-Exos could be internalized by GC-1 spg cells after I/R injury (Figure 6(a)). The western blotting results indicated that p-AKT and p-ERK1/2 expression was decreased in GC-1 spg cells subjected to I/R injury, while ADSC-Exos treatment activated the PI3K/AKT and MAPK/ERK1/2 pathways. Furthermore, pretreatment with LY294002 (PI3K/AKT inhibitor) and PD98059 (MAPK/ERK1/2 inhibitor) inhibited the expression of p-AKT and p-ERK1/2, respectively (Figures 6(b)–6(e)).

### 3.6. ADSC-Exos Regulate GC-1 spg Cell Proliferation and Migration

**Figure 7:**
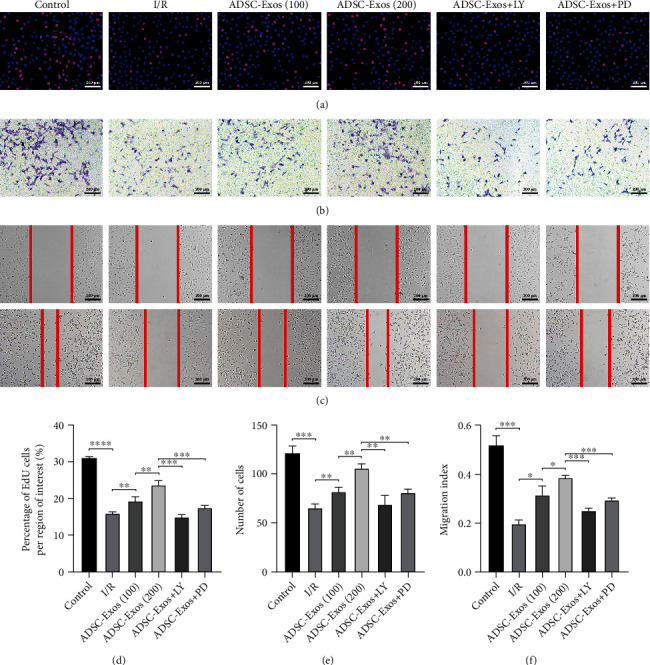
ADSC-Exos promote the proliferation and migration of spermatogenic cells after I/R injury. (a, d) Effect of ADSC-Exos on the proliferation of GC-1 spg cells by EdU assays. (b, c, e, f) Effect of ADSC-Exos on the migration of GC-1 spg cells by transwell assays and scratch test. Bars, 200 *μ*m. Data are represented as mean ± SD. ^∗^*P* < 0.05, ^∗∗^*P* < 0.01, ^∗∗∗^*P* < 0.001, ^∗∗∗∗^*P* < 0.001.

The effects of ADSC-Exos on the proliferation and migration of GC-1 spg cells were evaluated using different concentrations of ADSC-Exos, as well as pathway inhibitors. The results of the EdU assays showed that ADSC-Exos promoted GC-1 spg cell proliferation after I/R injury in a dose-dependent manner, whereas LY294002 and PD98059 significantly attenuated this effect (Figures 7(a) and 7(d)). Similarly, the results of the transwell assays (Figures 7(b) and 7(e)) and scratch tests (Figures 7(c) and 7(f)) showed that ADSC-Exos promoted GC-1 spg cell migration after I/R injury, which was suppressed by LY294002 and PD98059.

### 3.7. ADSC-Exos Protect GC-1 spg Cells against Apoptosis

**Figure 8:**
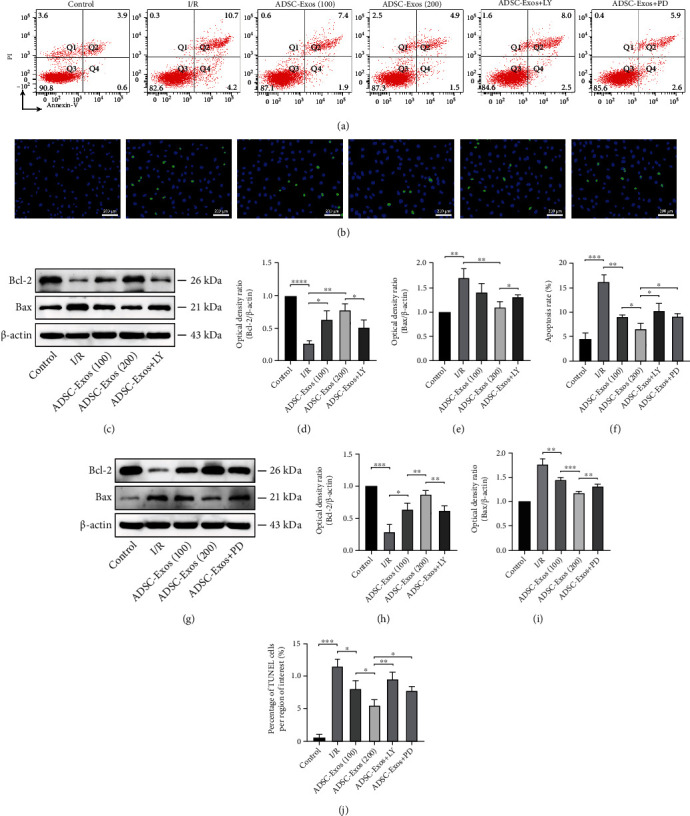
ADSC-Exos inhibit apoptosis of spermatogenic cells after I/R injury. (a, b, f, j) Effect of ADSC-Exos on apoptosis of GC-1 spg cells by flow cytometry analysis and TUNEL staining. (c–e, g–i) Western blot analysis of protein levels of Bcl-2 and Bax induced by ADSC-Exos or pathway inhibitors. Bars, 200 *μ*m. Data are represented as mean ± SD. ^∗^*P* < 0.05, ^∗∗^*P* < 0.01, ^∗∗∗^*P* < 0.001, ^∗∗∗∗^*P* < 0.001.

Flow cytometry and TUNEL assays were used to detect the apoptosis of GC-1 spg cells. The number of apoptotic GC-1 spg cells was significantly increased after I/R injury, whereas treatment with ADSC-Exos substantially reduced cell apoptosis. LY294002 and PD98059 inhibited the antiapoptotic effects of ADSC-Exos (Figures 8(a), 8(b), 8(f), and 8(j)). In addition, the western blotting results indicated that ADSC-Exos could increase I/R-induced low expression of Bcl-2 and decrease I/R-induced high expression of Bax. Similarly, LY294002 and PD98059 attenuated the regulation of Bcl-2 and Bax expression by ADSC-Exos (Figures 8(c)–8(e) and 8(g)–8(i)).

### 3.8. ADSC-Exos Regulate the Inflammatory Response Induced by Testicular Torsion-Detorsion Injury

**Figure 9:**
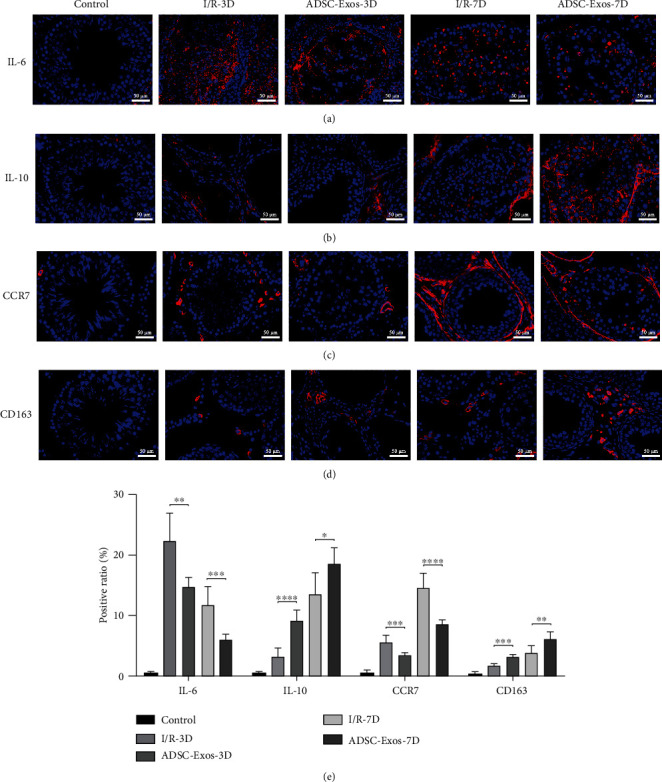
ADSC-Exos inhibit inflammatory expression induced by testicular torsion-detorsion injury. (a–d) The expressions of IL-6, IL-10, CCR7, and CD163 were detected by immunofluorescence at days 3 (*n* = 6) and 7 (*n* = 6). (e) Positive ratio of inflammation-related factors (*n* = 6). Bars, 50 *μ*m. Data are represented as mean ± SD. ^∗^*P* < 0.05, ^∗∗^*P* < 0.01, ^∗∗∗^*P* < 0.001, ^∗∗∗∗^*P* < 0.001.

Immunofluorescence analysis revealed that a large amount of IL-6 (proinflammatory factor) was aggregated within the testicular tissue on day 3 after testicular torsion-detorsion injury, which decreased on day 7 (Figure 9(a)). However, IL-6 expression was significantly decreased in the ADSC-Exos groups on days 3 and 7 compared to that in the I/R groups. In contrast, IL-10 (anti-inflammatory factor) expression was increased in the ADSC-Exos groups compared to that in the I/R groups (Figure 9(b)). In addition, the numbers of CCR7^+^ (M1 macrophage marker) and CD163^+^ (M2 macrophage marker) cells were increased after testicular torsion-detorsion injury. However, the ADSC-Exos groups had significantly fewer CCR7^+^ cells and significantly more CD163^+^ cells than the I/R groups (Figures 9(c) and 9(d)). The quantitative results are shown in Figure 9(e).

## 4. Discussion

Testicular torsion is a major cause of testicular loss in male adolescents [4]. Effective antioxidation and anti-inflammatory adjuvant therapy are the main means of reducing I/R injury after testicular torsion. Recent studies have shown that ADSC-Exos can effectively alleviate I/R injury of the cerebrum and myocardium [32]. In this study, ADSC-Exos were shown to reduce oxidative stress, inhibit inflammation, promote the proliferation and migration of spermatogenic cells, and prevent apoptosis in the testis.

The physiological properties of spermatogenic cells, which are borderline hypoxic, make them sensitive to changes in blood flow [33]. In the present study, rat testes that were severely damaged after torsion-detorsion injury exhibited seminiferous tubule disorder, interstitial edema, and few sperm. Tissue I/R injury is closely related to ROS. ROS production induced by torsion-detorsion injury exceeds the scavenging ability of antioxidant enzymes, leading to the accumulation of ROS in tissues. Subsequently, ROS production seriously damages spermatogenic and Sertoli cells, greatly affecting the spermatogenic function of the testes [34]. MDA is the end product of ROS and thus a reliable indicator of ROS levels [35]. SOD protects cells against superoxide radical damage by catalyzing the dismutation of superoxide radicals into H_2_O_2_ and O_2_ [36]. Several studies have shown that the application of antioxidants can reduce the level of oxidative stress in testicular tissue and improve the histological score of testes. Shokoohi et al. reported that *hesperidin* protected against oxidative damage caused by testicular varicocele in rats and reduced programmed cell death in germ cells [37]. Wei et al. demonstrated that probucol could effectively attenuate ROS overproduction induced by testicular torsion-detorsion injury and protect testicular spermatogenesis [38]. In addition, Zhou et al. found that transplantation of the uncultured adipose-derived stromal vascular fraction could promote spermatogenesis while reducing oxidative stress levels after testicular torsion [39]. Hsiao et al. obtained similar results using ADSCs in the treatment of testicular torsion-detorsion injury [18]. As expected, ADSC-Exos in the present study significantly reduced MDA levels, increased SOD levels, and improved sperm quality (quantity, morphology, and motility) after testicular torsion-detorsion injury.

After testicular torsion-detorsion injury, ROS production is accompanied by the activation of apoptotic pathways [40]. Apoptosis induced by testicular torsion-detorsion injury occurs in all spermatogenic cells, among which apoptosis of primary spermatocytes is the main reason for impaired fertility [41]. In the present study, TUNEL staining and Cleaved Caspase-3 immunofluorescence analysis indicated that after testicular torsion injury, the caspase-dependent apoptosis pathways were activated and spermatogenic cells were largely apoptotic, whereas treatment with ADSC-Exos significantly attenuated the degree of apoptosis in spermatogenic cells. Seven days after injury, the number of apoptotic cells was significantly decreased, indicating that the apoptotic spermatogenic cells had died. Similarly, Bai et al. reported that ADSC-Exos can effectively alleviate inflammation and apoptosis in skin flaps after I/R injury [42]. Moreover, Zhu et al. found that ADSC-Exos could protect renal tubular epithelial cells against apoptosis caused by renal I/R injury [43]. However, H&E staining in the current study indicated that the number of spermatogenic cells increased after ADSC-Exos treatment. Therefore, the effects of ADSC-Exos on the proliferation of spermatogenic cells were further investigated. Immunohistochemical analysis revealed that spermatogenic cell proliferation mainly occurred in primary spermatocytes in normal testicular tissues, which was inhibited after testicular torsion-detorsion injury. Following treatment with ADSC-Exos, the expression of Ki67 (cell proliferation marker) in spermatogenic cells was increased. Interestingly, the proliferative effect of ADSC-Exos on spermatogenic cells was not restricted to primary spermatocytes. These results suggest that ADSC-Exos can inhibit spermatogenic cell apoptosis and promote their proliferation after testicular torsion-detorsion injury.

Whether unilateral testicular I/R causes contralateral testicular damage is a controversial issue. Dejban et al. reported that unilateral testicular torsion significantly decreased spermatogenic function in the contralateral testis [44]. Wei et al. found that unilateral testicular torsion did not affect the contralateral testis [45]. In addition, Hsiao et al. detected no damage to the contralateral testis when rats were treated with ADSCs for unilateral testicular torsion [18]. Similarly, unilateral testicular torsion did not damage the contralateral testes in the current study. Compared with the Control and I/R groups, the contralateral testes in the ADSC-Exos groups showed no statistical difference in either spermatogenic function or sperm quality. Therefore, the study findings indicate that local injection of ADSC-Exos into the injured testis has no effect on the contralateral testis.

MicroRNAs, the main mediators of exosomal function [46], affect the expression of genes upon entering target cells, which in turn affects signaling pathways. In order to understand the mechanism underlying alleviation of testicular torsion-detorsion injury by ADSC-Exos, miRNA sequencing was conducted. Several identified miRNAs, including let-7c-5p, miR-143-3p, and miR-22-3p, have been previously shown to attenuate tissue I/R injury [47–49], which suggested that the therapeutic effect of ADSC-Exos may be synergistically mediated by multiple miRNAs. The results of the enrichment analysis of the top 50 miRNAs in ADSC-Exos indicated that the PI3K/AKT and MAPK pathways may play major roles. In addition, Lai et al. reported that the PI3K/AKT signaling pathway was involved in the therapeutic effects of ADSC-Exos in the treatment of cardiac I/R injury [50]. Meanwhile, Zhang et al. found that ADSC-Exos attenuated hepatic I/R injury *via* the MAPK/ERK1/2 signaling pathway [51]. Therefore, the association between ADSC-Exos and the PI3K/AKT and MAPK/ERK1/2 signaling pathways were examined *in vitro*. The results indicated that I/R injury inhibited p-AKT and p-ERK1/2 expression in GC-1 spg cells, while treatment with ADSC-Exos upregulated this expression. In addition, ADSC-Exos promoted proliferation and migration while inhibiting apoptosis of GC-1 spg cells after I/R injury. Pathway inhibitors LY294002 and PD98059 effectively inhibited the expression of p-AKT and p-ERK1/2, respectively, and blocked the protective effects of ADSC-Exos against GC-1 spg cell I/R injury. Taken together, these results support that ADSC-Exos alleviate testicular torsion-detorsion injury by promoting activation of the PI3K/AKT and MAPK/ERK1/2 signaling pathways.

The inflammatory response is an important pathological mechanism of I/R injury [52]. ROS-induced redox changes lead to the release of inflammatory cytokines [53]. Tamer et al. reported that the expression of TNF-*α* and IL-6 was increased after testicular torsion, while that of IL-10 was decreased [41]. Further, Turner et al. found that TNF-*α*, IL-6, and IL-1*β* released by macrophages exacerbated inflammation in testicular torsion-detorsion injury [54]. Recent studies have confirmed that polarization determines the role of macrophages in inflammation [55–57]. In addition, previous studies have shown that ADSC-Exos can modulate macrophage polarization from the proinflammatory M1 phenotype to the anti-inflammatory M2 phenotype *in vitro* [58–60]. In the present study, treatment with ADSC-Exos reduced the number of CCR7^+^ M1 macrophages and increased the number of CD163^+^ M2 macrophages after testicular torsion-detorsion injury. As expected, the expression of proinflammatory cytokine IL-6 was decreased after ADSC-Exos treatment, whereas that of anti-inflammatory cytokine IL-10 was increased. Therefore, ADSC-Exos may alleviate inflammation induced by testicular torsion-detorsion injury through modulation of macrophage polarization.

A previous study confirmed that testicular torsion for at least 1 h can cause substantial tissue damage [61], while torsion for more than 4 h reportedly causes irreversible focal infarction in testicular tissue [62, 63]. The current study is aimed at investigating the effects of local injection of ADSC-Exos on testicular torsion-detorsion injury without creating irreversible ischemic damage. Therefore, 3 h was selected to induce testicular torsion injury in the current study. Given the sudden and painful nature of testicular torsion, surgery is usually performed as soon as possible in the clinic. Thus, the reperfusion injury resulting from testicular detorsion is superimposed on the initial ischemic injury due to testicular torsion, resulting in secondary damage to spermatogenic cells. In order to prevent further injury due to reperfusion, pretreatment with ADSC-Exos before surgical detorsion is preferable to treatment after surgical detorsion. Additionally, considering that preoperative preparation usually lasts approximately 30 min, ADSC-Exos was locally injected into the testes 30 min before the detorsion procedure to improve the clinical relevance of the study findings. Moreover, because transplanted exosomes may not be able to enter the testis *via* normal circulation due to the physiological blood-testis barrier, ADSC-Exos were administered by local injection to the testis rather than typical intravenous administration. The low immunogenicity of exosomes, compared to that of stromal cells, enables their direct transplantation into organs such as the testis.

Nevertheless, this study had some limitations. First, the experiments were only performed in a rat model and have not been confirmed in humans. Thus, further clinical studies are needed to clarify the therapeutic effects of ADSC-Exos on human testicular torsion injury. Second, only the early efficacy of ADSC-Exos in ameliorating testicular torsion-detorsion injury was evaluated, and the long-term efficacy, such as fertility function, requires further study. Third, optimizing the dose and frequency of ADSC-Exos injections warrants further study. Finally, this study was based on global analysis of miRNAs in ADSC-Exos. Individual analysis of the identified miRNAs will be performed to determine the predominant miRNAs in subsequent research.

## 5. Conclusion

This study showed that ADSC-Exos can alleviate testicular torsion-detorsion injury by reducing oxidative stress and promoting M2 polarization to inhibit inflammation. ADSC-Exos activated the PI3K/AKT and MAPK/ERK1/2 pathways to promote the proliferation and migration of spermatogenic cells while inhibiting their apoptosis. In addition, this study provides a therapeutic reference for clinical use. Collectively, the study findings support the feasibility of using ADSC-Exos to protect against testicular torsion-detorsion injury and provide insight for future clinical treatment of testicular torsion.

## Figures and Tables

**Table 1:** Johnsen's score for spermatogenic function.

Score	Characteristics
10	Complete spermatogenesis with many spermatozoa
9	Many spermatozoa present but germinal epithelium disorganized with marked sloughing or obliteration
8	Only few spermatozoa (<5–10) present in the section
7	No spermatozoa but many spermatids present
6	No spermatozoa and only few spermatids (<5–10) present
5	No spermatozoa and no spermatids but several or many spermatocytes present
4	Only few spermatocytes (<5) and no spermatids or spermatozoa present
3	Spermatogonia are the only germ cells present
2	No germ cells but Sertoli cells are present
1	No cells in tubular section

## Data Availability

The datasets used and/or analyzed during the current study are available from the corresponding author on reasonable request.

## Supplementary Materials

Supplementary Materials Figure S1: the spermatogenesis in the contralateral testis. (a, b) H&E staining of contralateral testis after torsion-detorsion injury at day 7 (*n* = 6). (c–e) Results of sperm parameters (quantity, morphology, and motility) in the contralateral testis at day 7 (*n* = 6).Bars, 50 *μ*m. Data are represented as mean ± SD.
