# Supplementary figures and images for: Adipose Mesenchymal Stromal Cell-Derived Exosomes Prevent Testicular Torsion Injury via Activating PI3K/AKT and MAPK/ERK1/2 Pathways
Source: Oxid Med Cell Longev. 2022 Jun 16;2022:8065771. doi: 10.1155/2022/8065771 (PMC9225846; doi:10.1155/2022/8065771)

FIGURE S1


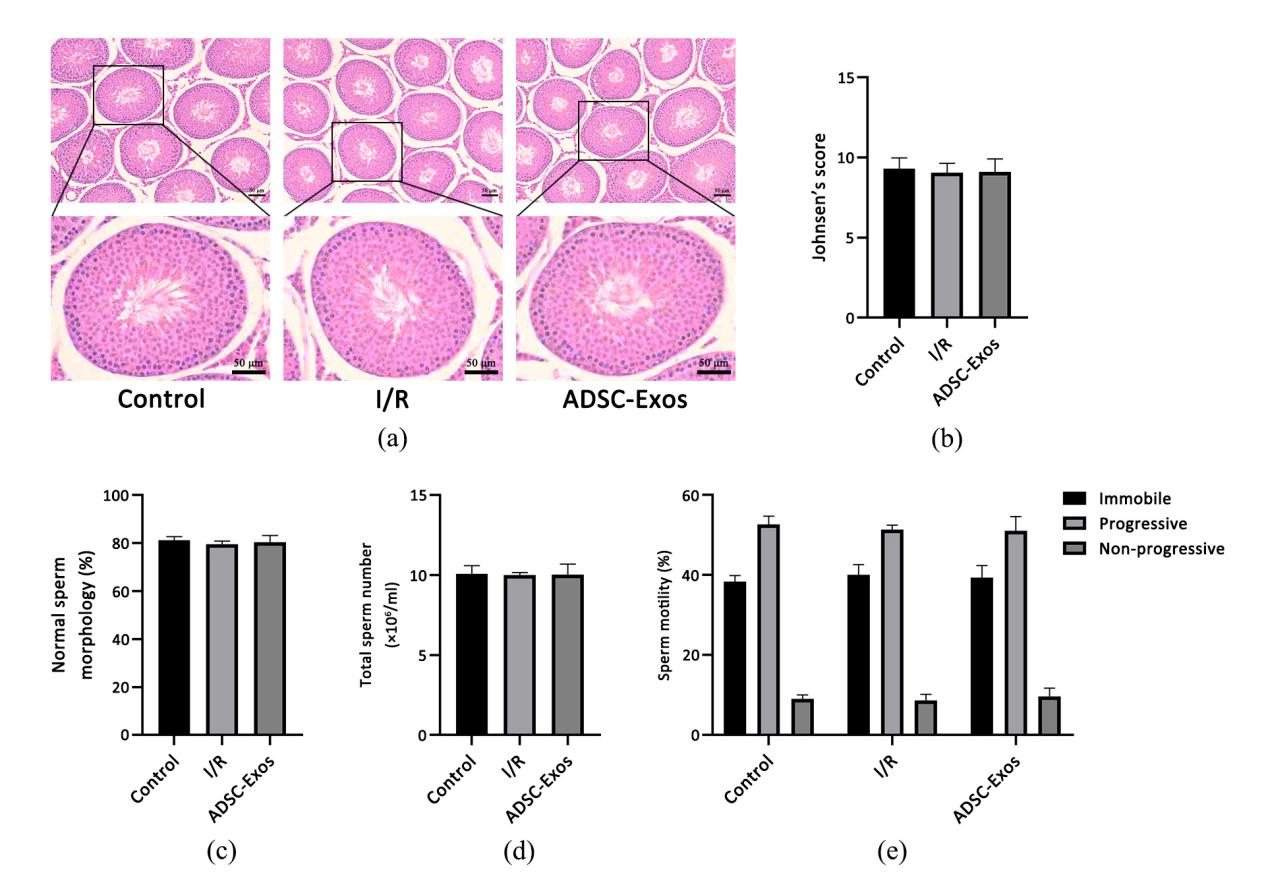


Graphical Abstract


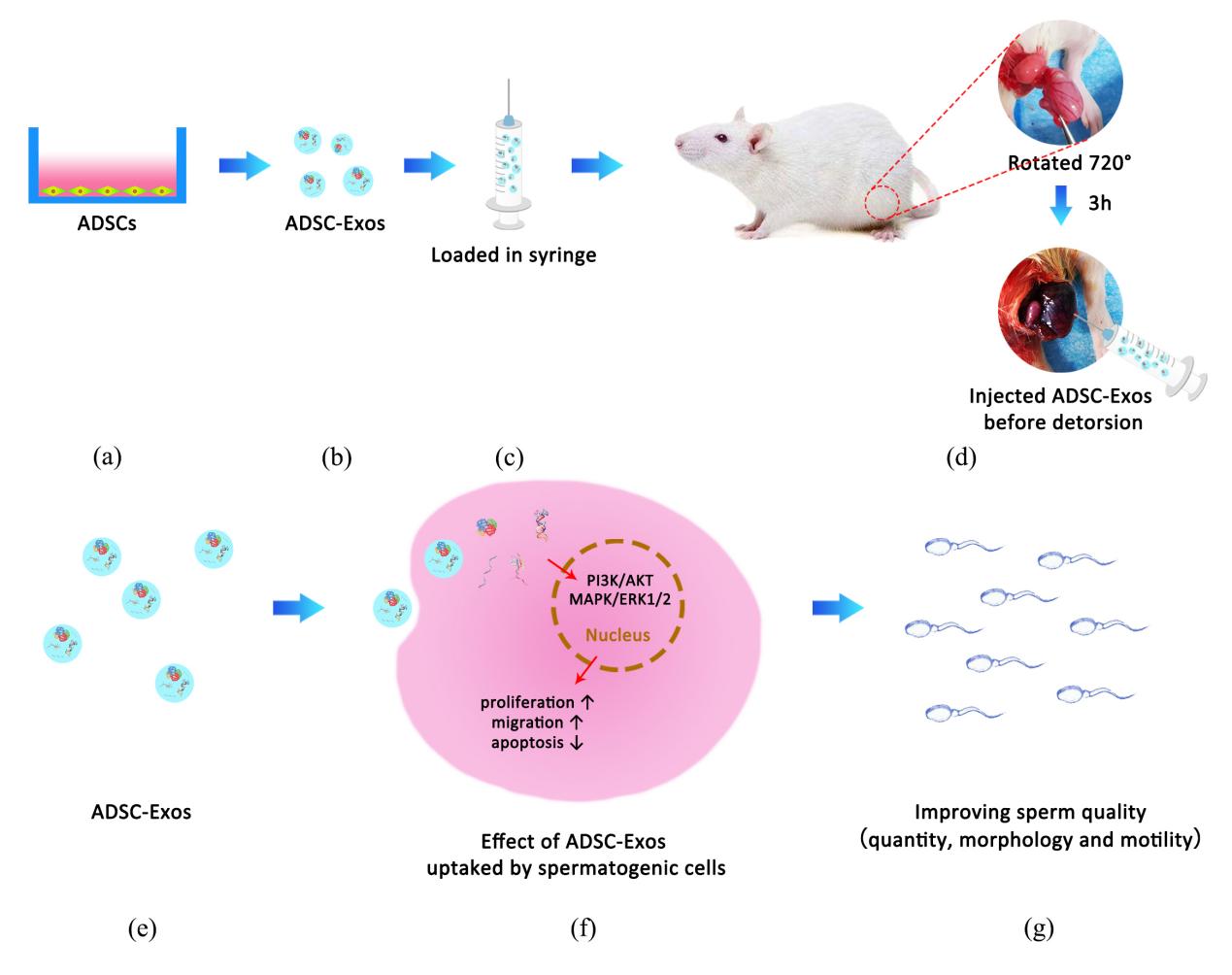

Supplement: Supplementary Materials — Figure S1: the spermatogenesis in the contralateral testis. (a, b) H&E staining of contralateral testis after torsion-detorsion injury at day 7 (n = 6). (c–e) Results of sperm parameters (quantity, morphology, and motility) in the contralateral testis at day 7 (n = 6).Bars, 50 μm. Data are represented as mean ± SD. [file 8065771.f1.docx]
